# Selenite inhibits glutamine metabolism and induces apoptosis by regulating GLS1 protein degradation via APC/C-CDH1 pathway in colorectal cancer cells

**DOI:** 10.18632/oncotarget.13600

**Published:** 2016-11-25

**Authors:** Junzhang Zhao, Rui Zhou, Kaiyuan Hui, Yang Yang, QiuYue Zhang, Yali Ci, Lei Shi, Caimin Xu, Fang Huang, Yu Hu

**Affiliations:** ^1^ Union Hospital, TongJi Medical College, Huazhong University of Science and Technology, Wuhan, China; ^2^ Department of Gastroenterology, Zhongnan Hospital of Wuhan University of Medicine, Wuhan, China; ^3^ The Hubei Clinical Center and Key Laboratory of Intestinal and Colorectal Diseases, Wuhan, China; ^4^ National Laboratory of Medical Molecular Biology, Department of Biochemistry and Molecular Biology, Institute of Basic Medical Sciences, Peking Union Medical College and Chinese Academy of Medical Sciences, Beijing, China; ^5^ Department of Gastroenterology, The Sixth Affiliated Hospital of Sun Yat-Sen University, Guangzhou, Guangdong, China; ^6^ Tumor Laboratory, Department of Radiation Oncology, The Affiliated Lianyungang Hospital of Xuzhou Medical University, Lianyungang, China

**Keywords:** glutamnase, selenite, colorectal cancer

## Abstract

Glutaminolysis is important for metabolism and biosynthesis of cancer cells, and GLS is essential in the process. Selenite is widely regarded as a chemopreventive agent against cancer risk. Emerging evidence suggests that it also has chemotherapeutic potential in various cancer types, but the mechanism remains elusive. We demonstrate for the first time that supranutritional dose of selenite suppresses glutaminolysis by promoting GLS1 protein degradation and apoptosis. Mechanistically, selenite promotes association of APC/C-CDH1 with GLS1 and leads to GLS1 degradation by ubiquitination, this process is related to induction of PTEN expression. In addition, GLS1 expression is increased in human colorectal cancer tissues compared with normal mucosae. Our data provide a novel mechanistic explanation for the anti-cancer effect of selenite from a perspective of cell metabolism. Moreover, our results indicate that glutaminolysis especially GLS1 could be an attractive therapeutic target in colorectal cancer.

## INTRODUCTION

Colorectal cancer is still a major cause of cancer death in the world giving that the rate decreases owing to colonoscopy and sigmoidoscopy screening [1]. In CRC patients, about half develop metastases, and most of these patients are deprived of opportunities for surgery [2, 3]. Additional treatment strategies are needed for these patients [2]. Altered cancer metabolic pathways represent attractive and promising therapeutic targets [4].

In order to satisfy the requirements of bioenergy and biosynthesis, metabolic machinery of cancer cells is re-programed [4]. Cancer cell metabolism re-programming involves several aspects, in which glycolytic pathway change is of critical importance. As a result of the glycolytic change, lactic acid, rather than acetyl-CoA is generated from pyruvate. Elevated glutamine metabolism in cancer cells has also been described, which can maintain a functioning citric acid cycle and then compensate for metabolic changes in cancer cells [5]. Glutamine (Gln), though generally considered as a non-essential amino acid in normal cells, is of key importance in proliferating cells and versatile in cancer cells [5, 6]. Glutamine can function not only as source of metabolic intermediates into TCA cycle, precursor for the biosynthesis of amino acids, glutathione and nucleic acids, but also supports the nitrogen-dependent anabolism [7]. Glutamine must be primarily converted to glutamate by glutaminase (GLS), which is prerequisite for the roles glutamine plays [6, 8]. Elevated expression of GLS1 was found in different tumor types and GLS1 activity inhibition could result in decreased growth rate of both tumor cells and xenografts tumors [9–11]. However, the mechanism by which GLS1 is regulated remains poorly understood. Ping Gao et al. demonstrated that c-myc increased GLS1 level via transcriptionally repressing miR-23a and miR-23b, which further led to increased glutamine metabolism [12]. K. Thangavelu et al. reported GLS1 activity was activated by EGF via Raf-Mek-Erk signaling module in a phosphorylation-dependent manner [13]. Recently, Shin et al. suggested a new molecular mechanism through which glutamate inhibited cell death by modulating a pathway involving MEK1, ERK2, GCN2, EIF2A, ATF4, TRB3, cFOS, and BID [14]. Nevertheless, regulation of GLS1 in different condition is rarely discussed.

Selenium, an essential metalloid trace element, is widely regarded as a chemopreventive and chemotherapeutic agent against multiple cancers [15, 16]. The inverse relationship between selenium and cancer risk has been proven by epidemiologic and preclinical data [17, 18]. Recently, it has been demonstrated by several studies that supranutritional dose of selenite could induce apoptosis in tumor cells of various origins *in vivo* and *vitro*, including lung cancer, prostate cancer, leukemia, brain glioma, breast cancer, cervical cancer and colorectal cancer [19–25]. Supranutritional dose of selenite are able to perturb cellular redox homeostasis by generating ROS (reactie oxygen species). Elevated ROS production exhibits its potent cytotoxic effects on proliferating cancer cells which have lower threshold tolerance to ROS contrast to normal cells [26–29]. Previous data suggest that by generating ROS, selenite targets several pivotal cancer-associated signaling pathways and induces multimodal regulated cell apoptosis, autophagy and mitophagy pathways [30–38]. Apoptosis can be induced by selenite through MAPK/PKD1/CREB/Bcl-2 pathway [33], PTEN/AKT/FoxO3a/Bim signaling pathway [32], RhoA/ROCK1/Erk1/2 pathway and AKT/b-catenin pathway [32, 36], and selenite induced autophagy through p70S6K/p53/ULK1 axis and PERK/eIF2a/ATF4 axis [38]. Cell cycle arrest could be induced by sodium selenite through ROS/JNK/ATF2 pathway and AKT/b-catenin pathway [35, 37]. However, the detailed molecular mechanisms by which selenite kills cancer cells remains elusive. Though poorly understood, previous studies proved that selenite could induce apoptosis of colorectal cancer cells [39].

Anaphase-promoting complex/cyclosome (APC/C) -CDH1 complex is a large multimeric ubiquitin ligase, which timely- and spatially-coordinated regulates cell cycle transitions, playing an key role in cells fate determination. Activation of APC/C requires the association of either Cdc20/fzy or Cdh1/fzr adaptor proteins, which recruit specific substrates containing certain motifs: D- and KEN-boxes [40].

Here in the study, we illustrated that selenite could suppress glutamine metabolism by degrading the key enzyme GLS1 in colorectal cancer cells and colon xenograft tumors. We firstly found that supranutritional doses of sodium selenite could repress GLS1 and upregulate PTEN, thereby promoting the binding of two ubiquitin ligases, anaphase-promoting complex/cyclosome–Cdh1 (APC/C-Cdh1). In addition, selenite induced degradation of GLS1 by ubiquitin occurred in nucleus rather than in cytoplasm in colorectal cancer cells. Taken together, our study demonstrated that sodium selenite regulates PTEN-APC/CDH1-GLS.

## RESULTS

### Upregulation of GLS1 in human colorectal cancer samples

**Figure 1:**
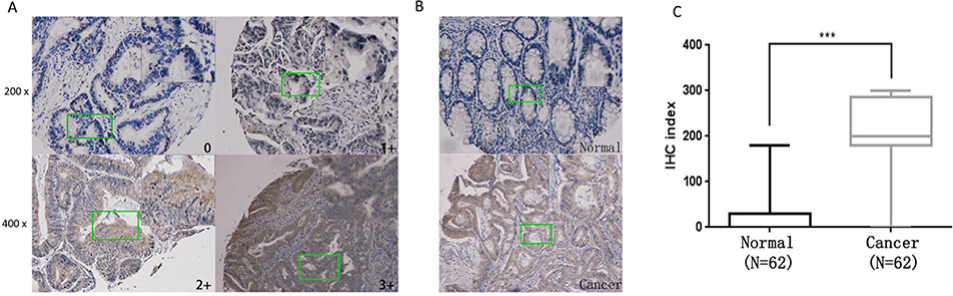
Upregulation of GLS1 in human colorectal cancer samples (**A**) Representative images of Immunohistochemistry (IHC) exhibiting negative (0), low (1+), moderate (2+) and high (3+) immunostaining for GLS1 protein from colorectal cancer and paraneoplastic tissue sections collected from 62 patients. 4 representative photomicrographs at × 100 magnification of sections tissue from patients with colorectal cancer were stained for GLS1. (**B**) Representative images of colorectal cancer (inferior panel) and normal tissues (upper panel) were stained for GLS1. (**C**) IHC exhibiting A box plot of GLS1 protein IHC index (score X % cancer cells) shows GLS1 protein IHC staining index significantly increased in colorectal cancer tissues in comparison with paraneoplastic tissues ( *P* < 0.001). A detailed summary of our IHC analysis is provided in Table 1 and clinicopathologic features of the patients is seen in Supplementary Table S1, respectively.

Increased expression of GLS1 in cell lines has been reported by several studies whereas few focuses on human colorectal cancer tissues. Here in our study, GLS1 was tested in 64 cancer and matched paraneoplastic normal tissues by immunohistochemistry. As indicated in Figure 1A, 1B representative staining is shown. Compared to paired paraneoplastic normal tissues, strong GLS1 cytoplasmic immunoreactivity is observed in some cancer tissues. Evaluation of staining positivity and intensity is seen in Material and Methods part, after exclusion of failure cases, 62 sections were scored and calculated. The result indicated that GLS positive staining was found in 61 cancer tissues and only 1 tumor cases were negatively staining while in 62 adjacent normal tissues, positive staining was only observed in 19 normal tissues. According to score of GLS1 expression, clinical data of 62 patients was collected and analyzed, the result showed that GLS1 score was positively correlated with TNM stage (Table 1) (*p* = 0.027). Thus, expression score of GLS1 was significantly elevated in colorectal cancer compared with adjacent normal tissues (Figure 1C) (*p* < 0.001).

**Table 1:** The relationships between the expression of GLS1 and clinicopathological features of colorectal cancer

Variable	GLS1 expression			*P* value
	None/low	Moderate	Strong	
Gender				0.814
Male	2	16	15	
Famale	3	14	12	
Age (Y)				0.632
< 60	1	6	3	
≥ 60	4	24	24	
TNM STAGE				0.027
I	1	6	1	
II	4	19	13	
III	0	5	12	
IV	0	0	1	
T STAGE				0.603
T1	0	2	0	
T2	1	3	2	
T3	3	23	22	
T4	1	2	3	
N STAGE				0.100
N0	2	23	12	
N1	2	6	11	
N2	1	1	4	
M STAGE				
M0	5	30	26	0.431
M1	0	0	1	

A level of *p* < 0.05 was considered significant.

### Selenite induces inhibition of glutaminolysis and downregulation of GLS expression

**Figure 2:**
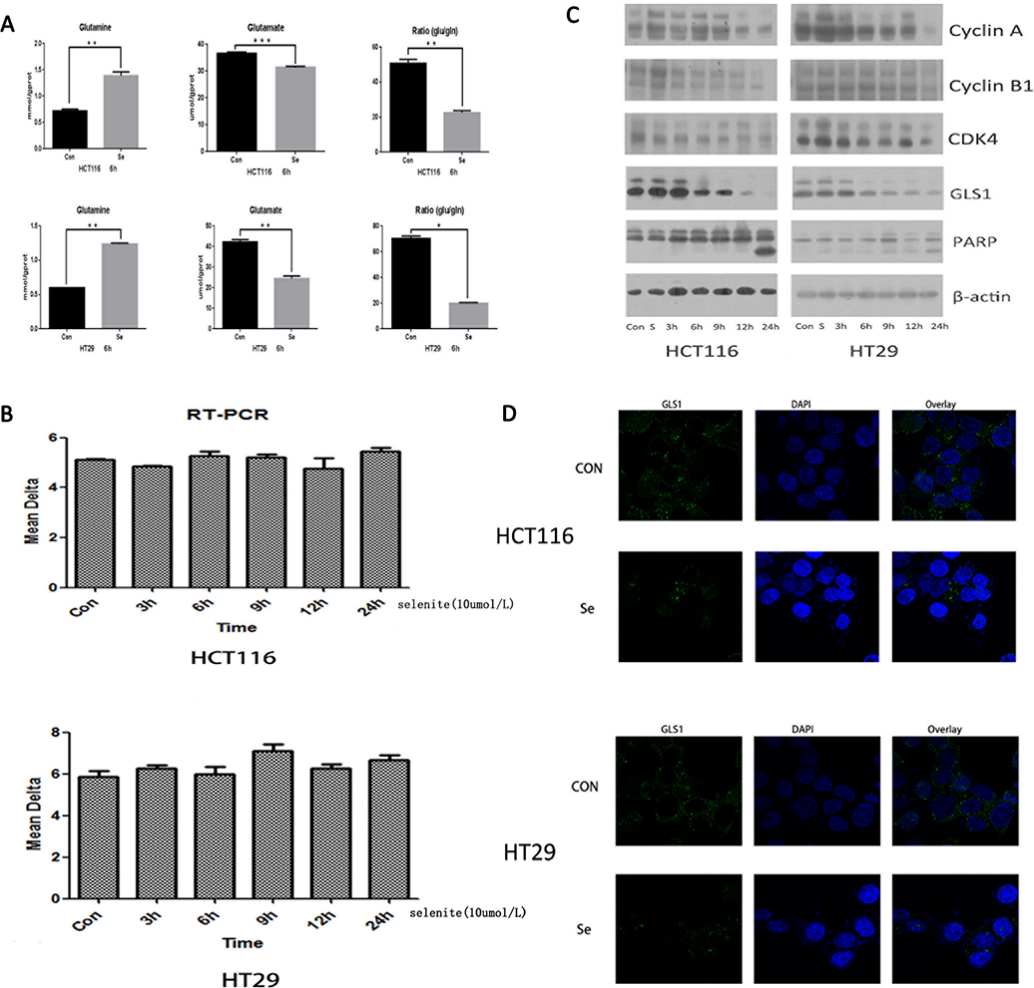
Selenite induces inhibition of glutaminolysis via downregulation of GLS1 expression (**A**) Selenite inhibited glutamine metabolism in CRC cells. Cells were treated with 10 umol/l selenite for 6 hours as indicated and then level of glutamine and glutamate in control and selenite-treated CRC cells was tested by Glutamine and Glutamate Determination Kit, the ratio of glutamate to glutamine was also shown. The glutamine level was significantly increased in selenite-treated CRC cells while both concentration of glutamate and ratio of glutamate to glutamine were significantly decreased in selenite-treated CRC cells in comparison with control cells. (**B**) Transcription of GLS1 is not significantly altered. HCT116 and HT29 CRC cells were treated with or without selenite for indicated time periods followed by reverse transcription PCR for three times. No significant difference was found. (**C**) Expression of GLS1 was decreased in CRC cells treated with selenite. Cells were treated with 10 umol/l selenite for various time periods and then immunoblotted for GLS1, cyclin A, cyclin B, CDK4, and PARP. B-actin was used as a loading control. (**D**) GLS1 protein in selenite-treated or control cells were immunostained with primary antibodies and the corresponding FITC-conjugated secondary antibodies followed by detection using confocal microscopy. Green signals indicated GLS1. Nuclei were counterstained with DAPI. Representative images of each sample are shown.

Apoptosis induction and cell cycle arrest of cancer cells by supranutritional doses of sodium selenite had been demonstrated by previous studies [35, 37]. We aimed to elucidate the detailed molecular mechanism, specially, from perspective of glutamine metabolism. Thus we test the alteration of glutamine and glutamate concentration in selenite-treated CRC cells by Glutamine and Glutamate Determination Kit. As indicated in the Figure 2A, compared with control groups, after treated with selenite for 6 hours, concentration of glutamine (gln) significantly increased while glutamate concentration and ratio of glutamate (glu) to glutamine decreased in both HCT116 and HT29 CRC cell lines. As known in the glutamine metabolism, glutaminase is the key enzyme responsible for catalyzing glutamine to glutamate. So, we conducted reverse transcription Polymerase Chain Reaction (RT-PCR) to exam the alteration of GLS1 transcriptional level in selenite-treated CRC cells, no significant difference was found in both CRC cells (Figure 2B). However, by performing western-blot, we found that GLS1 was time-dependently inhibited by supranutritional doses of sodium selenite in both HCT116 and HT29 CRC cells (Figure 2C), the result was also confirmed by immunofluorescence (Figure 2D). In addition, our result also suggested CRC cell cycle was arrested in G0/G1 phase (Figure 2C and Supplementary Figure S1), along with induction of apoptosis. Taken together, these results showed that sodium selenite suppressed glutamine metabolism by decreasing GLS1 level in HCT116 and HT29 CRC cells, which is not at transcriptional level.

### Selenite induces apoptosis via inhibition of glutaminolysis and GLS1 expression

**Figure 3:**
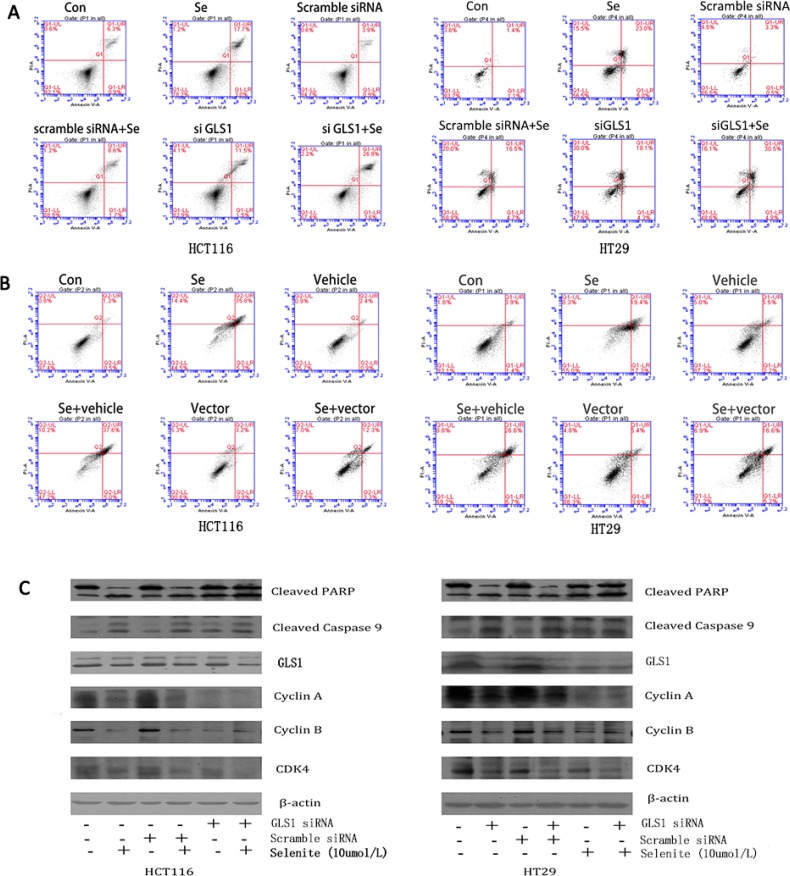

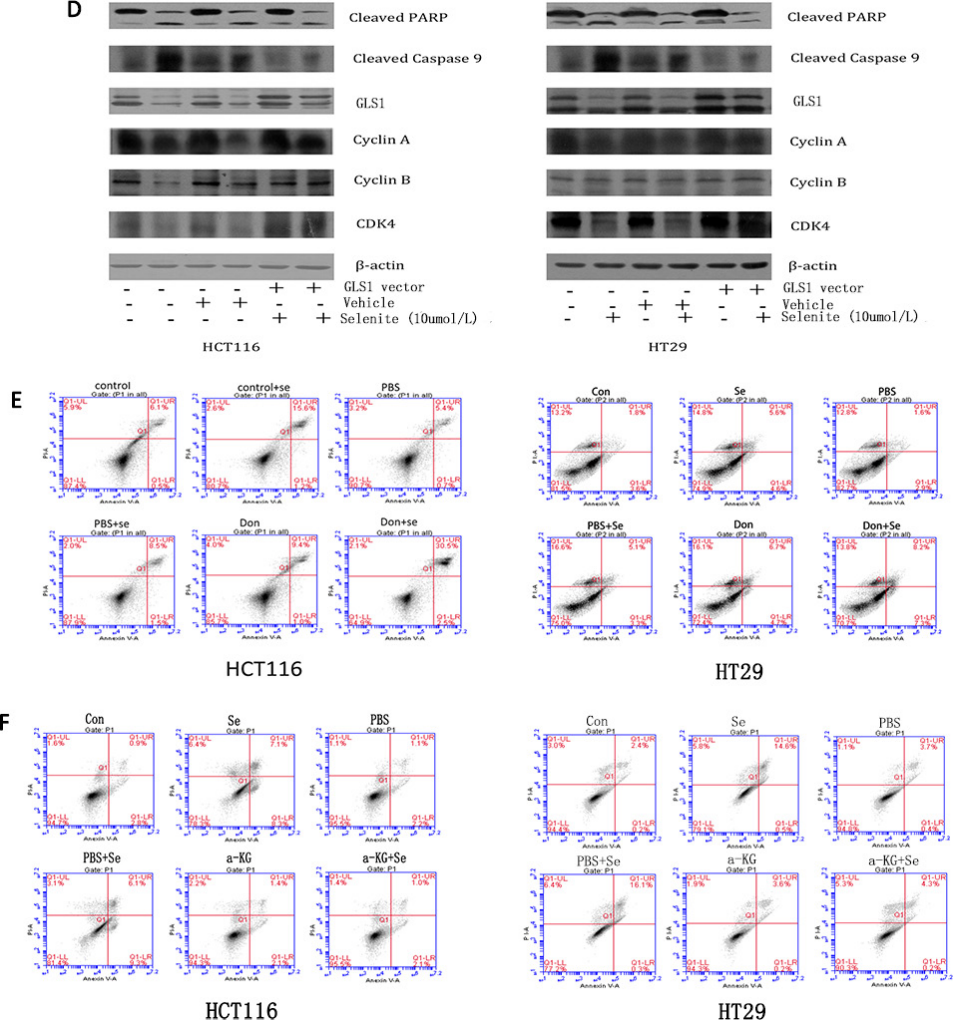
Selenite induces apoptosis via inhibition of glutaminolysis and GLS1 expression (**A**, **C**, **E**) Inhibition of GLS1 with either Don or GLS1 siRNA led to cell cycle arrest and increased apoptosis in selenite-treated HCT116 and HT29 CRC cells. Cells were treated with Don for 12 hours prior to selenite treatment or were transfected with GLS1 siRNA followed by treatment with either selenite or PBS for 24 hours. Cells were determined by FACS to analyze apoptosis rate or were then collected, and total cellular lysates were immunoblotted for cleaved PARP, GLS1, cleaved Caspase 9 and b-actin. (**B**, **D**) GLS1 overexpression protected cells from selenite-induced apoptosis. HCT116 and HT29 CRC cells were transfected with GLS vector prior to selenite treatment for 24 h and were then determined by FACS to analyze apoptosis rate or subjected to western blot assays using antibodies against cleaved PARP, GLS1, cleaved caspase 9. B-actin was probed to ensure equal protein loading. (**F**) a-KG reduced selenite-induced apoptosis in HCT116 and HT29 CRC cells. Cells pretreated for 1 hour with 10 umol/l a-ketoglutaric acid (a-KG), were treated with selenite (10 umol), apoptosis rate was analyzed by FACs.

Since selenite could induce apoptosis, cell cycle block, and suppression of glutamine metabolism, we next performed experiments to investigate whether inhibited glutamine metabolism was associated with selenite-induced apoptosis and cell cycle arrest in HCT116 and HT29 CRC cells. Don is reported to inhibit glutamine by suppressing glutamine utilizing enzymes activity while siRNA inhibits GLS1 expression level [10, 39]. As revealed in Figure 3C, expression of GLS1 was reduced by siRNA in selenite-treated CRC cells. Both GLS1 siRNA and Don treatments further significantly reinforced the selenite-induced apoptosis of CRC cells by flow cytometry (Figure 3A, 3E). Results from western-blot (Figure 3C) showed GLS1 inhibition led to more cleavage of apoptosis-related markers such as PARP and Caspase 9 in HCT116 and HT29 CRC cells and less cyclins such as cyclin A, cyclin B, CDK4 (Figure 3C) whereas GLS1 overexpression could largely eliminated the selenite-induced cell apoptosis and cell cycle arrest. Additionally, results of flow cytometry (Figure 3B) and western-blot (Figure 3D) demonstrated that in selenite-treated CRC cells, apoptosis rate decreased with GLS1 vector transfected. Furthermore, GLS1 inhibition resulted in a-KG deficiency and increasing apoptosis. When CRC cells were pretreated with a-ketoglutarate (10 Mm, PH was adjusted to 7.2) 2 hours [41], apoptosis induced by selenium was significantly suppressed in analysis with flow cytometry, as indicated in Figure 3F. In summary, these findings obviously demonstrated induction of cell cycle arrest and apoptosis in selenite-treated CRC cells was associated with glutamine metabolism suppression, especially through GLS1 inhibition and addition of a-KG reversed the results.

### Selenite promotes association of APC/C-CDH1 with GLS and leads to GLS degradation by ubiquitination

**Figure 4:**
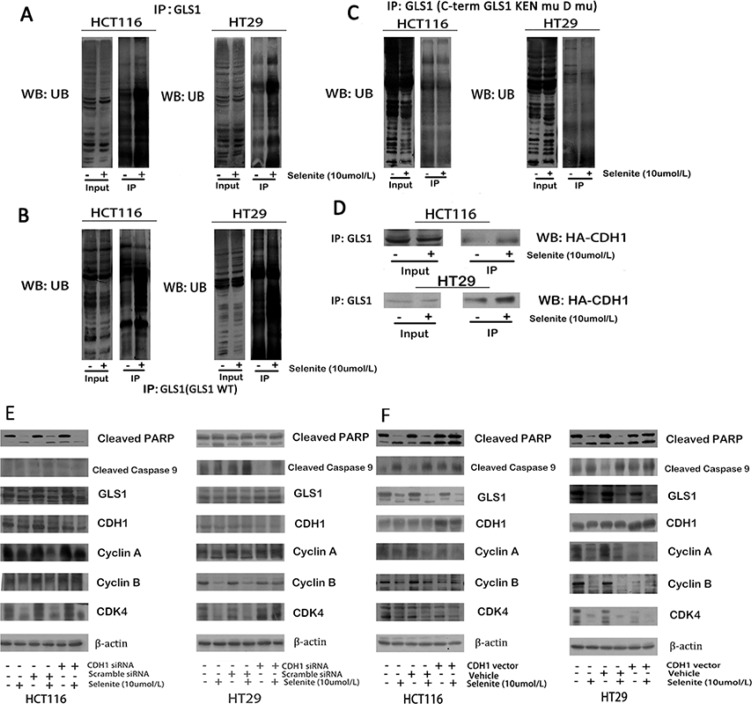
Selenite enhances GLS1 degradation by promoting association between CDH1 and GLS1 (**A**) Selenite (10 umol/l) treatment enhanced the interaction between GLS1 and ubiquitin. Ubiquitin was immunoprecipitated from selenite-treated and control cells by GLS1 antibody. The interaction between GLS1 and ubiquitin in the immunopreciptates was analyzed by western blot assay. All the blots were representative of three independent experiments. (**B**, **C**) APC/C-CDH1 recruited GLS by recognizing D box and KEN box, a prerequisite in selenite (10 umol/l) enhanced degradation of GLS. Cells were transfected with GLS wild-type or D box and KEN box double mutational vector prior to selenite treatment for 24 h and ubiquitin was immunoprecipitated from selenite-treated and control cells by GLS antibody. (**D**) Selenite enhanced the interaction between GLS and CDH1. Cells were transfected with HA-CHD1 vector prior to selenite treatment for 24 h and HA was immunoprecipitated from selenite-treated and control cells by GLS1 antibody. Selenite-enhanced PTEN modulated the APC/C-CDH1/GLS1 signaling pathway. (**E**, **F**) CDH1 promoted selenite-mediated degradation of GLS. Cells were transfected with CDH1 siRNA or CDH1 plasmids, then cells were treated with or without selenite for 24 h, and western blot was performed to analyze the expression levels of cyclin A, cyclin B, cleaved-PARP, cyclin D, GLS1, cleaved-Caspase. B-actin was used as a loading control.

Mechanism of decreased expression of GLS1 by supranutritional doses of selenite is not revealed in both HCT116 and HT29 CRC cells. We therefore performed a series of experiments to uncover how selenite triggers glutamine metabolism depression through suppressed GLS1. Sequence analysis revealed that GLS1 CDNA contained Lys-Glu-Asn box (KEN box) and a destruction box (D box) motifs, both of which could be targeted by Cdh1 [40]. After targeted by Cdh1, the GLS1-Cdh1 complex sequentially bind to the anaphase-promoting complex/cyclosome (APC/C), a large multimeric ubiquitin ligase, facilitating for further proteasomal destruction [40]. As indicated in Figure 4A, 4D, ubiquitination of GLS1 was enhanced after treated with supranutritional doses of selenite by co-immunoprecipitation, which was via promoted association with CDH1. Next, we carried out experiments to verify this hypothesis. Ubiquitination of GLS1 by CDH1 depended on the recognition sites, KEN box and D box, we modified both motifs by site-directed mutagenesis as described previously [40], resulting in destruction of recognition by CDH1. Co-expression of HA-CDH1 with double mutant GLS1 (GLS1 KEN^mut^ D box^mut^) caused obviously suppressed GLS degradation (Figure 4C), contrary to wild-type GLS1 (Figure 4B) transfected [40]. Additionally, in both HCT116 and HT29 CRC cells, knockdown of CDH1 partially rescued GLS expression (Figure 4E), along with relieved cell cycle arrest and apoptosis, and in contrast, and overexpressed CDH1 (Figure 4F) caused opposite effects. Thus, these results indicated selenite induced inhibition of glutamine metabolism was through ubiquitation of GLS1 by APC/C-CDH1 targeting both KEN and D boxes.

### Selenite enhances GLS ubiquitination by promoting PTEN expression

**Figure 5:**
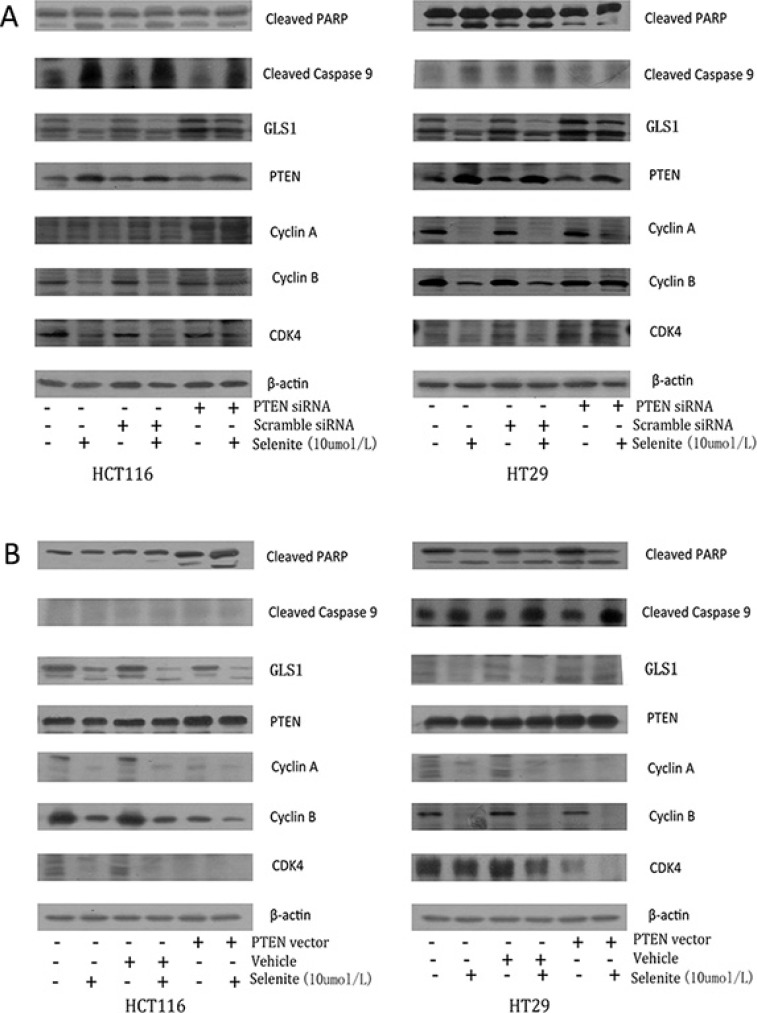
Selenite induces GLS ubiquitination by promoting PTEN expression (**A**) Cells were transfected with PTEN siRNA, then cells were treated with or without selenite for 24 hours, and the expression levels of cyclin A, cyclin B, cleaved-PARP, PTEN, GLS1 and cleaved-Caspase 9 were detected using western blotting. B-actin was used as a loading control. (**B**) Cells were transfected with wild-type PTEN plasmid, then CRC cells were treated with or without selenite for 24 hours, and detection of cyclin A, cyclin B, cleaved-PARP, PTEN, GLS1 and cleaved-Caspase 9 was performed by western blotting. B-actin was used as a loading control.

Previous work revealed that selenite enhanced increased PTEN expression via AKT/FoxO3a signaling pathway [32], and it has also been reported *in vivo* that nuclear localization of PTEN is necessary for activation of APC-CDH1, by which exerts its tumor-suppressive activity [42]. A series of experiments were performed to figure out the association of PTEN expression and GLS1 level in selenite-treated CRC cells. As seen in Figure 5, knockdown of PTEN resulted in decreased apoptosis markers (cleaved PARP and cleaved Caspase 9) and cyclins and CDK of CRC cells while selenite-induced CRC cell cycle arrest and apoptosis was enhanced with PTEN vector transfected.

Next, we tried to find the detailed mechanism in regulation of GLS1 by PTEN. Sergio L. Colombo et al. reported nuclear location of GLS1 was associated with its ubiquitination [42], though GLS1 is generally regarded located in cytoplasm and mitochondrial. Our findings proved GLS1, PTEN and CDH1 translocated to nuclear with selenite treated (Supplementary Figure S3A, S3C), and ubiquitination of GLS augmented in nuclear rather than in cytoplasm in both selenite-induced CRC cells (Supplementary Figure S3B). So, we speculated that nuclear accumulation PTEN enhanced ubiquitination of GLS1, which might occur in nuclear, and the detailed mechanism remains unclear. These results illustrated enhanced PTEN in selenite-treated CRC cells may contribute to degradation of GLS1, and this process may occur in nuclear.

### Induction of ROS contributes to suppression of glutamine metabolism by selenite

Our precious work has proven ROS level was elevated in selenite-treated CRC cells, which accounts for apoptosis and autophagy induction [30, 32, 38]. We then performed experiments to elucidate whether ROS is responsible for GLS1 inhibition in selenite-treated CRC cells. By using widely ROS scavenger MnTMPyP, a MnSOD mimic to eliminate ROS in selenite-treated cells, we can see from Supplementary Figure S2, depletion of ROS nearly completely relieved GLS1 suppression, along with cell cycle arrest and apoptosis abolished. While H2O2 triggered ROS generation, leading to the completely contrary effects. This suggested ROS played vital role in regulation of glutamine metabolism by suppressing GLS1 in selenite induced both HCT116 and HT29 CRC cells.

### Selenite induces PTEN/CDH1/GLS expression alteration and apoptosis in xenograft colorectal tumor model

Given that selenite induced glutaminolysis suppression in CRC cells, we next tested the effects of selenite on glutamine metabolism of CRC cells *in vivo* Colon xenograft tumor model was established by inoculating HCT116 CRC cells into 4-week-old immunodeficient nude mice subcutaneously. Mice were randomly divided into three groups, then intraperitoneally injected with PBS or different dose of selenite (1 mg/kg/day or 2 mg/kg/day) every other day when tumours were detected. After treatment with selenite for 3 weeks, mice were sacrificed and analyzed. For initial *in vivo* toxicity studies, 1 mg/kg/day or 2 mg/kg/day of selenite intraperitoneal injection did not result in a significant histopathological change in the major internal organs (liver, spleen and kidneys) between the vehicle-treated and selenite-treated groups [30, 32]. Tumour growth was significantly attenuated with selenite treated(Inhibition rate: 40.5% at 1 mg/kg/day and 51.4% at 2 mg/kg/day, *P* < 0.01, both ), yet no adverse effects occurred in body weight or activity [30]. Additionally, DNA fragmentation was increased *in situ* in selenite-treated HCT116 xenograft tumours by TUNEL assay [31].

**Figure 6:**
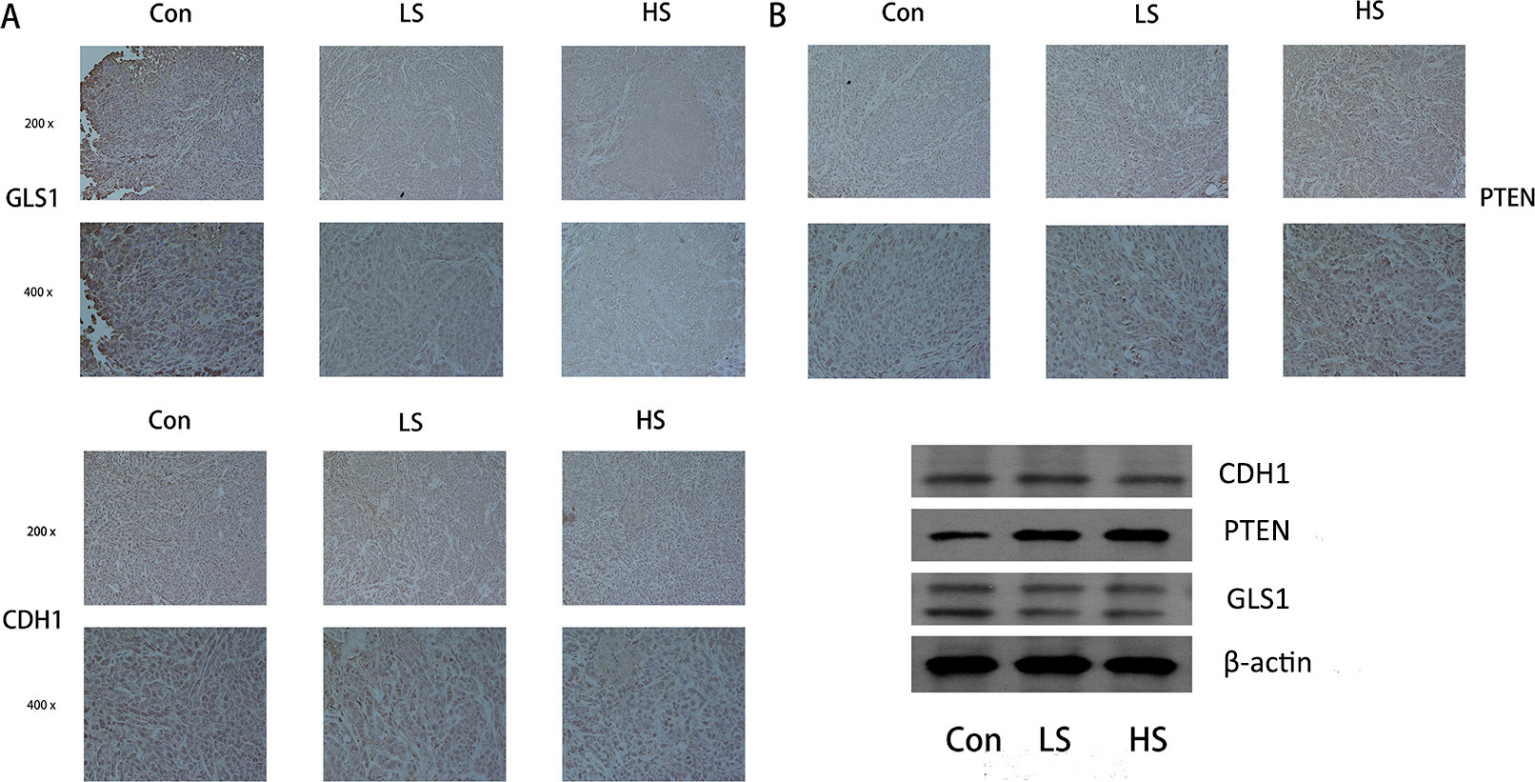
Selenite regulated the PTEN/APC/C-CDH1/GLS signaling pathway *in vivo* (**A**) Immunohistochemistry was performed in tumor tissues from a colon xenograft animal model using antibodies against PTEN, CDH1 and GLS. Representative images from control, low dose (LS) (8 umol) of selenite-treated samples, high does (HS) (15 umol) of selenite-treated samples are indicated. (**B**) Proteins extracted from xenograft tumor tissues were analyzed by Western blot using antibodies against critical molecules as shown.

Our previous work illustrated that by different pathways selenite induced apoptosis and inhibited tumor growth in SW480 and HCT116 colon xenograft model [30–32, 37]. We next carried out experiments to test whether glutaminolysis suppression could be induced by selenite *in vivo*. By performing western blot analysis of tissues from control, low dose (1 mg/kg/day), medium dose (1.5 mg/kg/day) and high dose (2 mg/kg/day) selenite-treated samples, we demonstrated that PTEN enhanced degradation of GLS by selenite also occurred *in vivo* (Figure 6B). Additionally, immunohistochemistry experiments were carried out to test expression of critical molecular involved in PTEN/APC/C-CDH1/GLS1, including PTEN, CDH1 and GLS1 (Figure 6A). We found that these molecules followed a similar pattern with those in CRC cell lines.

## DISCUSSION

It has been known for over half a century that tumors characterized an elevated demand for nutrients so as to satisfy their rapid proliferation, in which consumption of glutamine is of importance and enhanced in cancer cells [43–46]. A-ketoglutarate (α-KG) derived from metabolism of glutamine is multifunctional in the TCA cycle: it is not only as a major source of energy generates, generating reducing equivalents for the electron transport chain (ETC) and oxidative phosphorylation, but also serving as a key anaplerotic nutrient, which supply anabolism by feeding net production of oxaloacetate to offset export of intermediates from the cycle [47]. Though pleiotropic roles of glutamine plays in cancer metabolism, it must be firstly deamidized to glutamate by GLS, a prerequisite for the entry of TCA cycle [9]. GLS1, as the key enzyme in glutamine metabolism, is a promising and potential therapeutic target giving the facts that: a) glutamine is unnecessary for normal cells while essential for cancer cells *in vivo* and vitro [10], b) activity or expression of GLS1 was activated or increased in several tumor origins and inhibition of activity or depression of GLS1 expression resulted in decreased proliferation rate in cancer cells [12, 14, 40] and c) oncogenes and tumor suppressor genes are involved in regulation of GLS1 [13, 48, 49].

The chemopreventive and chemotherapeutic effects of selenite has been well validated in colorectal cancer by epidemiologic and pre-clinical studies [50], but the underlying mechanisms still remains elusive. It is proven that both selenoproteins and low molecular weight selenium metabolites contributed to anti-cancer effect of selente [51, 52]. Ip et al. showed that methylated selenium metabolites exhibit greater protection against cancer than compounds converted to support selenoprotein synthesis [53]. Previous studies have proved that selenite is a strong oxidizing agents and reacts with thiols [54]. Classical reaction by Painter et al. demonstrated that selenious acid reacts with thiols to form disulfides and mixed selenium trisulfides: 4 RSH + H2SeO3→RSSR + RS-Se-SR + 3 H2O [55], while generation of selenide is under anaerobic conditions with consumption of three molecules of NADPH, along with complete 6-electron reduction: SeO_3_^2−^ + 3 NADPH + H^+^ →Se^2−^ + 3 NADP^+^ 3 H_2_O [54]. Selenide, selenodiglutathione (GS-Se-SG) and monomethylselenol react with thiols, creating redox cycles, resulting in thiol oxidation and the formation of ROS [54]. Our previous results revealed that in CRC cells, ROS increased in a time-depend manner with selenite treated [37]. ROS is a critical step in selenium-mediated cytotoxicity in several tumor forms, including colon, prostate, lung, and bone. The biological effect of selenium metabolites in cancer cells were investigated extensively, including apoptosis [56], senescence [57], mitophagy [58], endoplasmic reticulum stress [59] and so on. We report for the first time that selenite suppresses colorectal cancer glutamine metabolism especially targeting GLS1 expression both *in vitro* and *in vivo* [10], providing a novel explanation for the anti-cancer effect of selenite. Results also showed that after treated with selenite for 6 hours, discrepancy occured between the changes of glutamine and glutamate concentration. We speculated this dues to proteasomal recycling of proteins provides Glu, or autophagy provides Glu under the circumstances, and further study is still needed to the hypotheses. Elevated expression or activity of GLS1 in various cancer types were reported in series of studies [11, 46, 47–49] , but few focused on human colorectal cancer tissues [60]. In our study, increased expression of GLS1 is validated in human colorectal cancer compared with paired adjacent normal tissues [60]. In addition, our previous study reported SLC1A5, an important glutamine transporter, was also upregulated in human colorectal cancer tissues [61], suggesting extensively activation of the glutamine metabolism pathway. We also provided evidence that supranutritional dose of selenite induced cell death in colorectal cancer cells without affecting normal intestine epithelial cells [31], which is consistent with the observation in this study that selenite retarded xenograft colorectal tumor growth without affecting the body weight of mice. Taken together, these results provided reasonable assumption that selenium compound could blunt upregulated glutamine metabolism in colorectal cancer with minimal adverse effect to normal cells, which needs further corroboration in more sophisticated *in vivo* models.

Recent studies showed that GLS was subjected to extensive oncogenic control. C-myc is among the most frequently reported oncogene, Gao et al. reported that c-myc transcriptionally represses miR-23a and miR-23b, leading to overexpression of GLS1 [12]. GLS1 was also reported to be regulated by series of oncogene or tumor-suppressors including Rho GTPase, ERBB2, EGFR, most have bearing on transcriptive regulation [13, 46–48]. PTEN is one of the most frequently mutated tumor-suppressors in many sporadic and heritable tumor types [53], and estimated frequency of monoallelic mutations at PTEN varies from 30% to 50% in colorectal cancer [63]. Our previous study showed that selenite induces increased expression of PTEN, which further potentiates apoptosis through inhibition of the PI3K/AKT/FoxO3a signaling pathway in colorectal cancer cells [32]. In this paper, we found we found that increased expression of PTEN inhibited glutaminolysis pathway by promoting GLS1 degradation. These evidence indicate that selenite restore the tumor-suppressive function of PTEN to exert anti-cancer effects in colorectal cancer. Another kind of glutaminase, GLS2, also catalyzes the hydrolysis of glutamine to glutamate [9]. Previous studies demonstrated that level of p53 remained unchanged with selenite treated, whereas phosphorylated p53 significantly decreased [64]. Though p53 is involved in regulation of GLS2, expression of GLS2 remained almost the same in selenite-treated CRC cells (results were not shown), further researches are needed to uncover the detailed mechanism.

APC/C-CDH1 complex is an important downstream effector of PTEN, which timely- and spatially-coordinated degrades cell cycle regulators and regulates cell cycle transitions [65]. Activation of APC/C requires the binding of either Cdc20/fzy or Cdh1/fzr adaptor proteins, two of which recruit specific substrates containing motifs: D- and KEN-boxes [66]. PFKFB3 and GLS1, both contain D box and KEN box, could be targeted by APC/C-CDH1 [34]. Furthermore, recent findings expand APC/C-CDH1 function to genomic integrity, signal transduction, cell differentiation and tumorigenesis [67]. Aberrant Cdh1 activity or ablation of Cdh1 deprives its role of tumor suppression, thus contributes to carcinogenesis and other diseases [67, 68]. In selenite-treated colorectal cancer cells, We showed that selenite promotes binding of APC/C-CDH1 with GLS1, which targeted GLS1 for further degradation. After mutation of D box and KEN box in GLS plasmid, degradation of GLS1 was largely eliminated, which indirectly suggested that GLS1 was degraded at post-transcriptional level targeted by APC/C-CDH1. Currently, there are potential compounds targeting GLS including 968 and BPTES, all of them disrupt the enzyme activity of GLS1. In addition, our results provide evidence that GLS could be regulated in a post-translational way, and APC/C-CDH1 might be a potential target.

## MATERIALS AND METHODS

### Patients and tissue microarray (TMA)

64 fresh tumour and paraneoplastic specimens from colorectal cancer patients were collected from the Union Hospital of Tongji Medical College of Huazhong University of Science and Technology (Hubei, China) as approved by the Human Ethics Review Board.

All subjects were informed and signed the aggrement of sample collection. Furthermore, colorectal cancer tissues were obtained, which included 64 pairs of tumour and matched normal colonic tissue in total. The diagnosis of specimens was confirmed by immunohistochemistry, and all of the patients were staged in accordance with the 7 Th American Joint Committee on Cancer (AJCC) stages.

### Reagents and antibodies

Sodium selenite, Tiron, buthionine-sulfoximine (BSO) were purchased from Sigma-Aldrich (St. Louis, MO, USA). MG-132 was purchased from Selleckchem (Houston, USA). MnTMPyP was obtained from Merck Calbiochem (San Diego, CA, USA). DAPI was from Beyotime (Haimen, Jiangsu, China). Glutamine and Glutamate Determination Kit was purchased from Nanjing Jiancheng Bioengineering Institute (Nanjing, Jiangsu, China).

The antibody recognizing b-actin, cyclin A, cyclin B, CDK4 and B23 was purchased from Santa Cruz (Santa Cruz, CA, USA). Antibodies against cleaved PARP cleaved Caspase 9, PTEN, cyclin D1 and HA-tag were purchased from Cell Signaling Technology (Beverly, MA, USA). Antibodies against GLS1 and APC3 were purchased from Abcam (Cambridge, UK). Antibodies against CDH1 was obtained from Abengt (San Diego, CA, USA).

### Cell lines and culture

Human colorectal cancer cell lines HCT116 and HT29 were obtained from the cell culture center of the Institute of Basic Medical Sciences, Chinese Academy of Medical Sciences and grown in DMEM (Hyclone, Logan, USA) supplemented with 10% fetal bovine serum (Hyclone, Logan, USA), 1% penicillin and streptomycin at 37°C under 5% CO2. All cell lines were discarded in 2 months and changed to new lines propagated from the frozen stocks and cell lines were monitored routinely during the period.

### SDS-PAGE and western-blot

SDS–PAGE and western blot assay were carried out as described previously. In briefly, total cell were lysed and suspended in RIPA buffer (Beyotime, Haimen, China). Total proteins were obtained by sonication and centrifugation as described previously. Nuclear fractions were obtained by using nuclear/cytoplasmic extraction kit (Beyotime, Haimen, China). Protein concentration was determined by Bradford assay. Subsequently, equal aliquots of cell lysates were subjected to 10% or 12% SDS–PAGE and immunoblotted with different primary antibodies as described above, then corresponding HRP-conjugated secondary antibodies. The visualization of immunoreactive bands was performed by chemiluminescence assay in accordance with the manufacturer's recommendations (Thermo Fisher, Waltham, MA, USA).

### Plasmids and transient transfection analysis

The human cDNA GLS1 was presented as a gift from Prof. David Piwnica-Worms at MD Anderson, which was inserted into pCDNA v5/6-His. The Site-directed mutagenesis of the KEN box and D box in Gls1 was performed according to the manufacturer's instructions of Quick Change site-directed mutagenesis kit (Stratagene, La Jolla, CA, USA), as described previously. Mammalian expression vector encoding human CDH1 was obtained from Cathie M. Pfleger (Massachusetts General Hospital Cancer Center, Charlestown) and PTEN was from Maria-Magdalena Georgescu (MD Anderson Cancer Center, USA).

Indicated plasmids were transfected into cells by lipofectamine 2000 (Invitrogen Paisley, Scotland, UK) in accordance with the instructions. In brief, to allow for cell attachment and growth, one day before transfection, approximately 4 * 10^5^ cells were seeded into six-well plate. Approximately 4 ug plasmids were transfected into cells with 50% confluency by 5 ul lipofectamine 2000 regent per well. 24 hours later, cells were treated with selenite or solution control ((Phosphate Buffered Solution, PBS).

### Small interfering RNAs

GLS siRNA (ON-TARGET plus SMARTpool target sequences are 5′-CCUGAAGCAGUUCGAAAUA-3′, 5′-CUGAAUAUGUGCAUCGAUA-3′, 5′-AGAAAGUG GAGAUCGAAAU-3′ and 5′-GCACAGACAUGGUUG GUAU-3′); PTEN siRNA (5′-GACUUGAAGGCGU AUACAGtt-3′); CDH1 siRNA (5′-GAAGAAGGGUCU GUUCACGtt-3′; 5′-GGAACACGCUGACAGGACAtt-3′) and control siRNA (5′-UUCUCCGAACGUGUCA-CGUTT-3′) were chemically synthesized by GenePharm (Shanghai, China). In brief, approximately 4 * 10^6^ cells maintained in 6-well plate were ransfected with 100 pM siRNA using lipofectamine 2000 according the protocol as described above. Then as demanded, cells were subjected to further treatment.

### Co-immunoprecipitation

Cells were trypsinized and harvested, then lysed in RIPA lysis buffer on ice for 30 mins. Afterwards, protein supernatant was obtained by centrifugation at 12000 rpm at 4°C for 15 mins. Appropriate antibodies were used to immunoprecipitate protein lysates (200 ug) and normal immunoglobulin antibodies were used as a control. 25 ul protein A + G agarose beads (Santa Cruz) were used to capture the immunoprecipitates. The immunoprecipitates were wushed and elutioned for further western blot assays.

### RT-PCR

Total RNA was extracted by Trizol agent (Invitrogen, Carlsbad, CA, USA) following the manufacturer's instructions. Total RNA samples were reversely transcripted by 5 × All-In-One RT MasterMix (abmGood, Richmond, BC, Canada). The expression of target gene was normalised to GAPDH gene expression level. Real-time PCR was carried out with UltraSYBR Mixture (With ROX) (CWBIO, Beijing, China) on Bio-Rad IQ5 (Bio-Rad, Hercules, CA, USA). Primers for GLS1 (Pari1: forward: 5′-AGTGACTTGTGAATCAGCCAG-3′, reverse: 5′-GT TGCCCATCTTATCCAGAGG-3′; Pair2: forward: 5′-GCT GTGCTCCATTGAAGTGA-3′, reverse: 5′-GCAAACTG CCCTGAGAAGTC-3′) and primers for GAPDH (forward: 5′-CATCTTCCAGGAGC-GAGATC-3′, reverse: 5′-GCTTGA-CAAAGTGGTCGTTG-3′) were synthesized by Tsingke Biological Techology (Beijing, China).

### Apoptosis assay

Annexin V/PI double staining with the Apoptosis Detection Kit (Merck Calbiochem, USA) was used to evaluate the percentage of cells undergoing apoptosis. The assay was carried out following the manufacturer's instructions. In brief, after the indicated treatments, cells were trypsinized and harvested, which were then washed twice with pre-cold PBS buffer, and afterwards, Annexin V and PI in 1 binding buffer were used to stain cell. The stained cells were analysed by Accuri C6 flow cytometer (Accuri Cytometers Inc., Ann Arbor, MI, USA). All experiments were carried out three times independently, and the results are shown as the mean values ± S.D.

### Immunofluorescence

Cells were fixed on a coverslip and stained with primary antibodies (1:50), then with secondary antibodies (1:200) which were conjugated with FITC or CY3 (Jackson ImmunoResearch Laboratories, PA, USA) followed by counterstaining with DAPI solution. Cell images were captured with an Olympus laser scanning confocal FV1000 microscope (Olympus, Tokyo, Japan) and analysis of acquired images was performed by Olympus Fluoview software (Tokyo, Japan).

### Immunohistochemical staining

The establishment of colorectal xenograft model was described previously [26, 29]. All animal procedures were carried out according to the guidelines issued by the committee on animal research of Peking Union Medical College and approved by the institutional ethics committee. The section of both the control and selenite-treated tumor tissues groups was performed at the termination of the experiments. Half of these samples were embedded in paraffin for immunohistochemical analysis whereas the remaining tissues were homogenized and subjected to western blotting analysis.

Immunohistochemistry analyses were performed to access GLS1 expression in 62 pairs of both fresh tumour and paraneoplastic specimens in colorectal cancer patients, while expression of GLS1, PTEN and CDH1 were detected in both the control and selenite-treated xenograft tumor tissues. Formalin-fixed paraffin-embedded tissue sections were deparaffinized in xylene and rehydrated in graded alcohol. Endogenous peroxidase activity was blocked with 3% hydrogen peroxide in methanol for 10 min. Antigen retrieval was performed by autoclave sterilization in sodium citrate buffer for 3 min. In order to reduce background non-specific staining, slides were incubated with 10% normal goat serum solution for 20 min. The rabbit polyclonal antibody against GLS1, PTEN and CDH1 was applied at a concentration of 1:100 and incubated at 4°C overnight. HRP-conjugated secondary antibody was performed according to the manufacturer's instructions. The slides were then incubated with DAB to visualize GLS1, PTEN and CDH1 expression and followed by hematoxylin counterstaining.

The images were captured using a RGB JVC solid-state camera connected to an Olympus BH2 microscope at 10- and 20- fold objective magnification fitted with a motorized stage. Immunohistochemical analysis of GLS1, PTEN and CDH1 was done according to standard procedures. Staining results were assessed by two pathologists independently.

### Evaluation of immunohistochemical staining

Immunohistochemical analysis of GLS expression was performed in accordance with standard procedures in a blinded fashion. Staining results were assessed by two pathologists independently with no knowledge of patients’ characteristics. The expressions of GLS1 was accessed semiquantitatively based on proportion and staining intensity of positive stained cells. Proportion of GLS1 was scored using semiquantitative criterion: 0 (no staining); 1, minimal (< 10%); 2, moderate (10–50%); and 3, diffuse (> 50%) positive cells. Staining intensity of GLS was also classified as 0 (negative); +1 (weak); +2 (moderate); and +3 (strong). These two scores, composed of both proportion and staining intensity, were added to give each case the sum score from 0 to 6, and according to the sum scores, cases were categorized to give final expression scores as – (0), negative; 1+ (1 or 2), weakly positive; 2+ (3 or 4), moderately positive; 3+ (5 or 6), strongly positive.

### Statistical analysis

Values are shown as means ± S.D. Statistical analysis was carried out using a software package (SPSS, version 19.0, Chicago, IL, USA). Analysis of the clinic pathological features and data were carried out with Person's Chi-Square and Likelihood Ratio tests. A level of *p* < 0.05 was considered significant.

## SUPPLEMENTARY MATERIAL FIGURES AND TABLE


