# Supplementary material for: Selenite inhibits glutamine metabolism and induces apoptosis by regulating GLS1 protein degradation via APC/C-CDH1 pathway in colorectal cancer cells
Source: Oncotarget. 2016 Nov 25;8(12):18832–47. doi: 10.18632/oncotarget.13600 (PMC5386651; doi:10.18632/oncotarget.13600)
Supplement: Supplementary file 1 [file oncotarget-08-18832-s001.pdf]

Selenite inhibits glutamine metabolism and induces apoptosis by regulating GLS1 protein degradation via APC/C-CDH1 pathway in colorectal cancer cells

Supplementary Materials

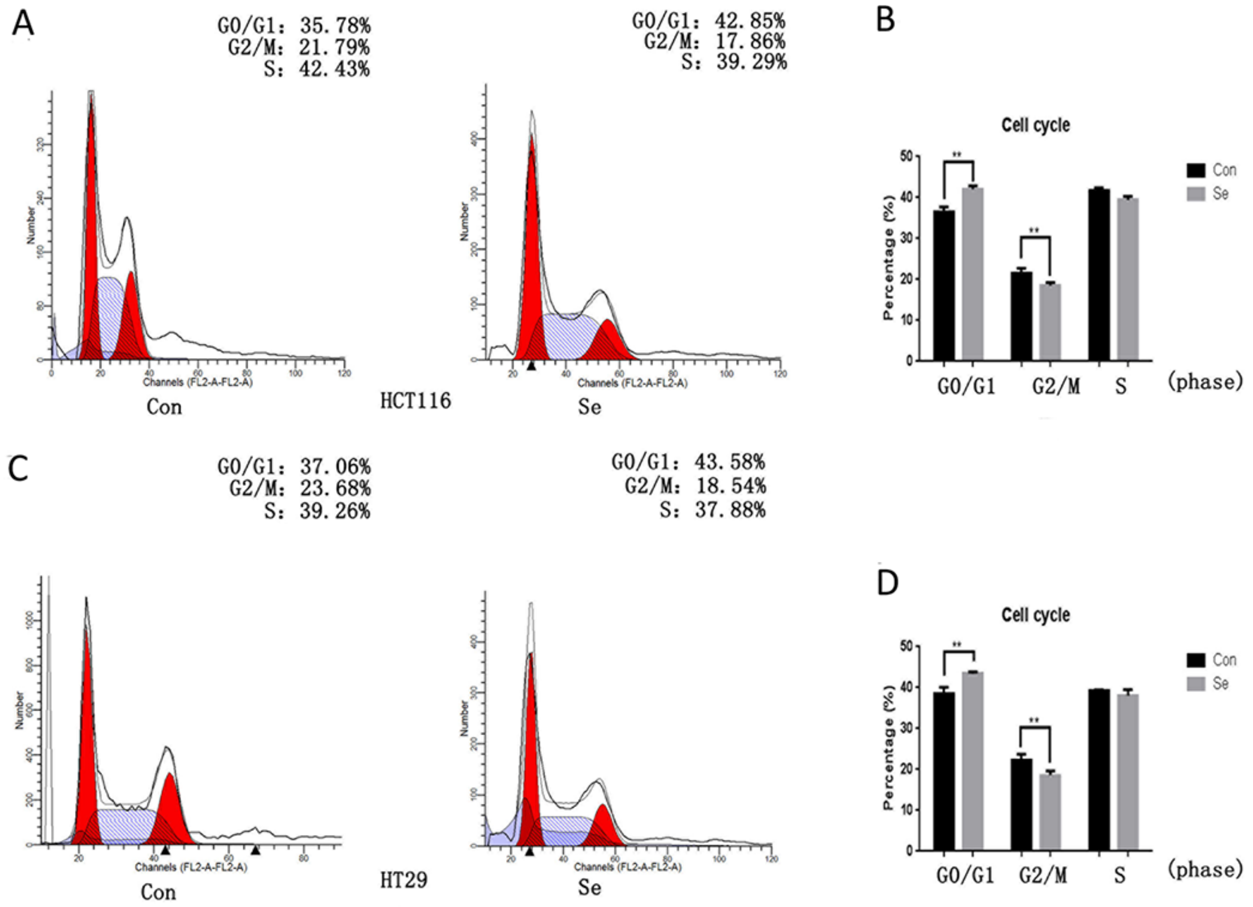

Supplementary Figure S1: HCT116 and HT29 CRC cells in the G0/G1 phase. (**A**, **C**) After treatment with 10 umol/L doses of selenite, cells for 24 hours, cells were evaluated by flow cytometric analysis. (**B**, **D**) The bar graphs represented the percentages of cells, and the experiments were repeated at least three times. $^{*}P < 0.05$, $^{**}P < 0.01$.

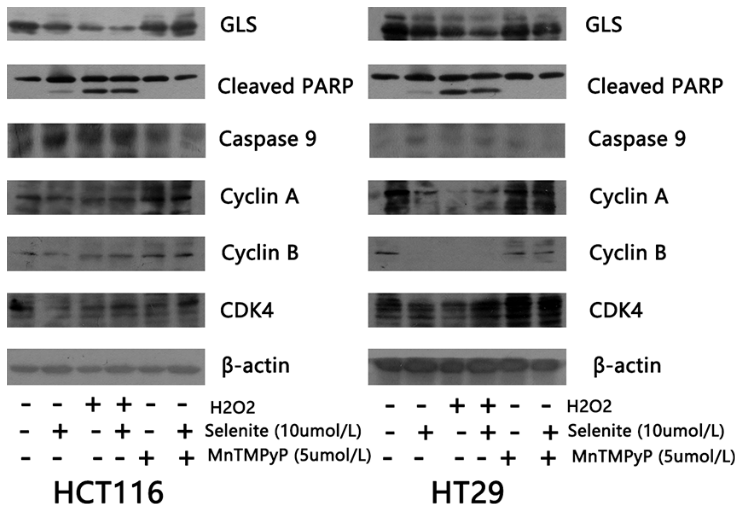

Supplementary Figure S2: Induction of ROS by selenite are involved in PTEN enhanced inhibition of glutamine metabolism. HCT116 and HT29 cells were pretreated with MnTmPyP or H2O2 solution and then were treated with selenite or PBS for 12 hours. Western blot was performed for analysis of the molecules indicated in the figures.

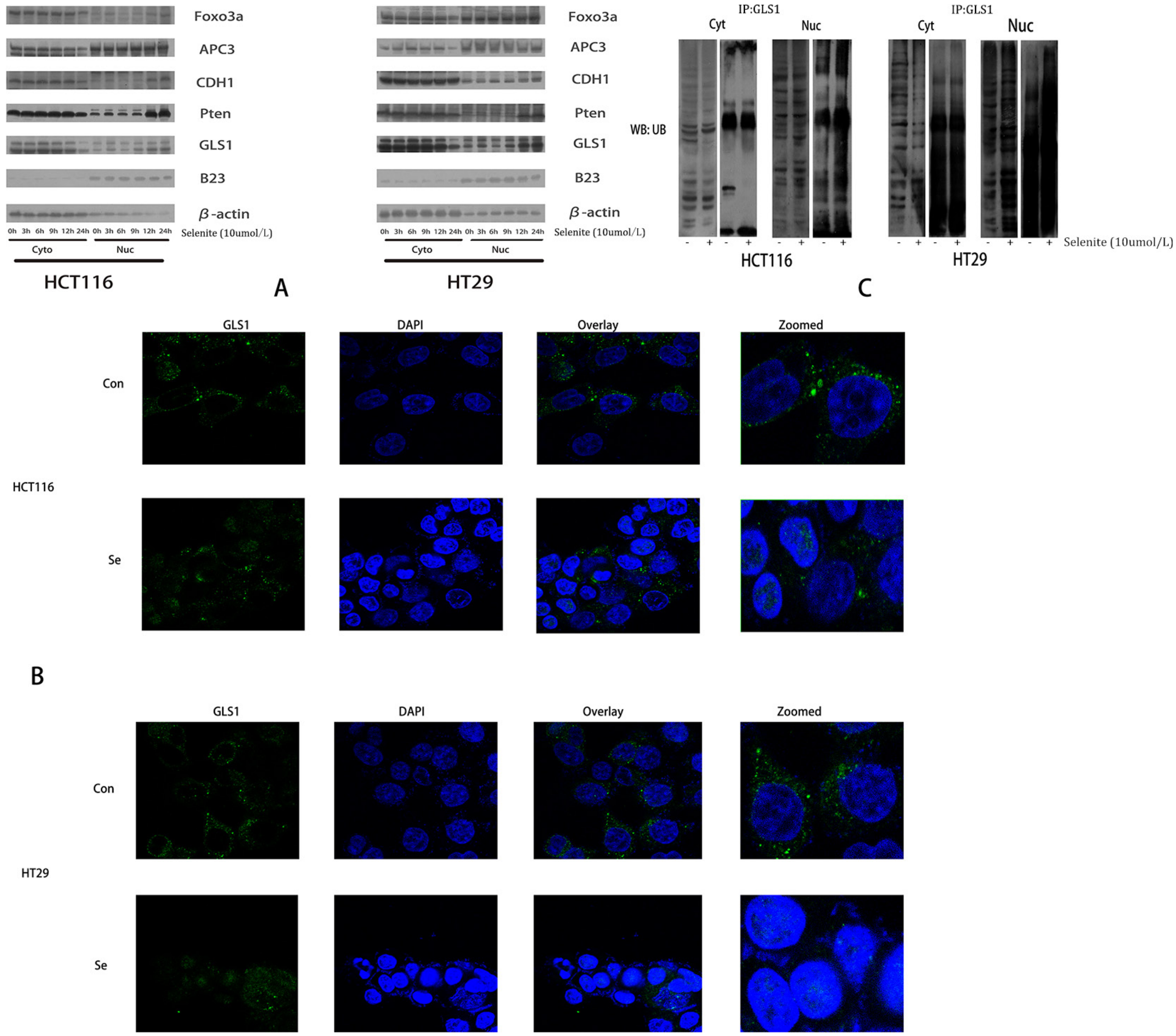

Supplementary Figure S3: Selenite-induced GLS translocation to nuclear contributes to its ubiquitination. (Selenite-induced GLS1 translocation to nuclear contributes to its ubiquitination. (**A**) Selenite treatment caused the translocation of GLS1, PTEN, CDH1, APC3 and FOXO3a from the cytoplasm to the nuclear. HCT116 and HT29 CRC cells were treated with selenite for the indicated time periods, and the nuclear were then isolated and immunoblotted for GLS, PTEN,CDH1, APC3 and FOXO3a. B23 and b-actin were used as markers of the nuclear and cytoplasm, respectively. (**B**) GLS1 was translocated from cytoplasm to nuclear with selenite treated. GLS1 protein in selenite-treated or control cells were immunostained with primary antibodies and the corresponding FITC-conjugated secondary antibodies were used to immunostained in selenite-treated or control cells, which were then detected by confocal microscopy. Green signals indicate GLS1. Nuclei were counterstained with DAPI. Representative images of each (**C**) Selenite enhanced ubiquitation of GLS1 occurred in nuclear rather than in cytoplasm. HCT116 and HT29 CRC cells were treated with selenite (10 umol/l) for 24 hours, cytoplasm and nuclear were separated, ubiquitin were immunoprecipitated by GLS1 antibody.

Supplementary Table S1: Clinicopathologic features of the patients

Variables	Results
**NO. of patients**	62
**Age, y (median, range)**	65.73/39–87
**Gender (male/female)**	33/29
**TNM Stage (I + II + III + IV)**	8/36/17/1
**T stage (T1 + T2/T3/T4)**	8/48/6
**N stage (N0/N1 + N2)**	37/19/6
**M stage (M0/M1)**	61/1
**Pathology (Tubular adenocarcinoma/Others)**	62/0

Clinicopathologic information of 62 patients were collected and analyzed, including age, gender, TNM stage and pathology.
